# Association between governmental spending on social services and health care use among low-income older adults

**DOI:** 10.1093/haschl/qxae181

**Published:** 2025-01-10

**Authors:** Carlos Irwin A Oronce, Ninez A Ponce, Catherine A Sarkisian, Frederick J Zimmerman, Yusuke Tsugawa

**Affiliations:** Division of General Internal Medicine and Health Services Research, Department of Medicine, David Geffen School of Medicine, University of California, Los Angeles, Los Angeles, CA 90024, United States; Department of Medicine, VA Greater Los Angeles Healthcare System, Los Angeles, CA 90025, United States; Department of Health Policy and Management, Jonathan and Karin Fielding School of Public Health, University of California, Los Angeles, Los Angeles, CA 90095, United States; Center for Health Policy Research, University of California, Los Angeles, Los Angeles, CA 90024, United States; Division of General Internal Medicine and Health Services Research, Department of Medicine, David Geffen School of Medicine, University of California, Los Angeles, Los Angeles, CA 90024, United States; Geriatrics Research Education & Clinical Center, VA Greater Los Angeles Healthcare System, Los Angeles CA 90025, United States; Department of Health Policy and Management, Jonathan and Karin Fielding School of Public Health, University of California, Los Angeles, Los Angeles, CA 90095, United States; Division of General Internal Medicine and Health Services Research, Department of Medicine, David Geffen School of Medicine, University of California, Los Angeles, Los Angeles, CA 90024, United States; Department of Health Policy and Management, Jonathan and Karin Fielding School of Public Health, University of California, Los Angeles, Los Angeles, CA 90095, United States

**Keywords:** social determinants of health, dual-eligibles, social policy, healthcare utilization

## Abstract

Prior research demonstrates that local government spending on social policies, excluding health care, is linked to improved population health. Whether such spending is associated with better access to primary care and reduced acute care utilization remains unclear. In this cross-sectional study, we evaluated the associations between county-level social spending and individual-level health care utilization among low-income Medicare beneficiaries, aged ≥65 years, from 2016 to 2018. We linked claims data to 4 categories of county-level government expenditures from the US Government Finance Database, including (1) public welfare, (2) public transit, (3) housing/community development, and (4) infrastructure-related social services. The main outcomes were annual primary care visit rates, emergency department visits, and preventable hospitalizations. After adjusting for patient and county characteristics, beneficiaries living in counties with higher spending on housing/community development had 11% higher primary care visit rates. Additionally, those living in counties with higher public transit and housing/community development spending experienced 6%-10% lower preventable hospitalization rates. Lower preventable hospitalization rates were especially pronounced among acute conditions. These findings suggest that investments in social services that address the health-related social needs of low-income older adults may be an important factor to consider in population-level efforts to reduce acute care utilization.

## Introduction

Across the United States, there are wide and growing income-related disparities in population health. For example, the gap in life expectancy for low-income adults varies as much as 15 years based on residence.^1,2^ Despite the increasing attention in health care on the unmet social needs and risk factors of individual patients and how these shape disparities, there has been relatively less work dedicated to understanding the distal policy factors outside of health care that influence patient outcomes.^3,4^ Some evidence suggests that higher government spending on social programs is associated with improved health across multiple indicators, including mortality, asthma, severe maternal morbidity, and low birthweight.^5-16^ Social spending encompasses a wide range of categories, beyond means-tested assistance programs targeted to individuals, and includes spending that shapes the environment in which health happens, such as housing, transportation, and other core services related to physical environments and infrastructure. However, the extent to which these investments contribute to improved access to care and reduced acute care utilization remains unclear, hindering our understanding of the potential returns on social services investment.

One plausible mechanism is that social spending increases the accessibility of timely primary and preventive care, for example, by reducing transportation barriers. Addressing these barriers, especially for low-income populations, may facilitate access to medical care and therefore reduce the need for costlier forms of health care, such as hospitalization.^17-20^ Another pathway, independent of access to primary care, is that social spending may improve the built and physical environments in which health is shaped. For example, government spending for building inspections and waste management may reduce exposures to environmental hazards (eg, mold, poor air quality) and thereby reduce acute care use (emergency department [ED] visits and hospitalizations).^21-24^ Given the growing policy attention on expanding primary care, reducing costly acute care, and addressing the drivers of worse population health, quantifying the relationship between patterns of social spending and health care use has important implications.

This is particularly salient for vulnerable, low-income older adults, who live on fixed monthly budgets that are often supplemented by safety-net assistance programs. As a result, they are susceptible to rising housing costs and frequently rely on public transit for everyday tasks and attending medical appointments.^25,26^ Low-income older adults in Medicare, who are dually eligible for Medicaid, experience a greater burden of chronic disease, greater limitations in activities of daily living (eg, dressing oneself), and subsequently, higher hospitalization and mortality rates compared to the non-dual population.^27,28^ Prior work has also demonstrated that better access to primary care for this population is associated with lower rates of preventable hospitalizations.^29^

In this context, the objective of this study is to test the associations between local social spending levels at the county level and the use of primary care, ED, and inpatient services among low-income older adults enrolled in Medicare.

## Data and methods

### Study data and sample

**Figure 1.**
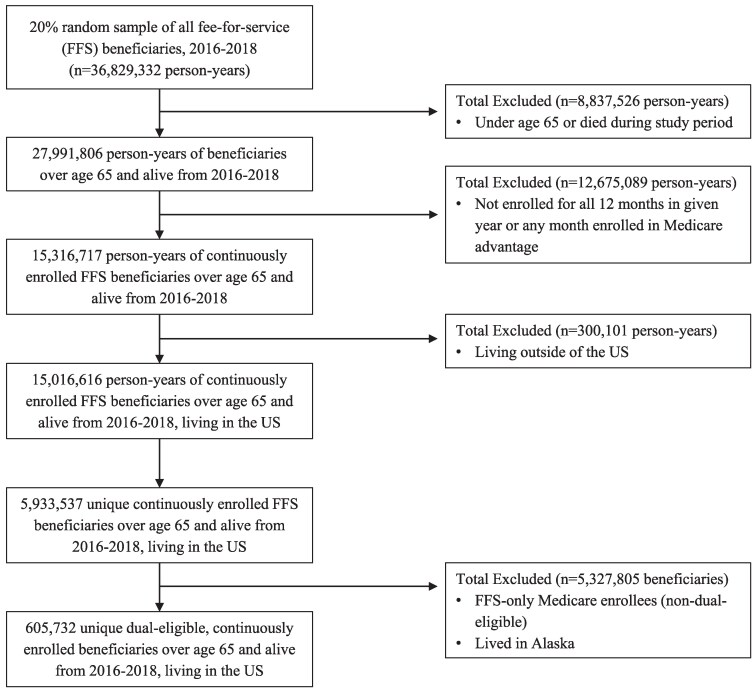
The study flow diagram outlines the starting population during 2016-2018 and the application of inclusion/exclusion criteria to arrive at the final study population.

For this cross-sectional study, the study population consisted of a 20% random sample of Medicare fee-for-service beneficiaries from 2016 to 2018. Beneficiary demographic characteristics and monthly enrollment status were obtained from the Medicare Master Beneficiary Summary File and linked to inpatient, outpatient, and carrier claims files. We included beneficiaries 65 years and older who were continuously enrolled in both Part A and Part B for at least 1 year and were alive throughout the year. We excluded those with any month of enrollment in Medicare Advantage or residence outside of the US. Consistent with prior work, we identified Medicare beneficiaries as low-income on the basis of Medicaid enrollment for 1 or more months in the year (Figure 1).^27^

We linked beneficiary-level data to county and zip code-level measures from the US Government Finance Database and the Area Health Resources File (AHRF) using the county federal information processing system code of the beneficiary. The US Government Finance Database compiles cleaned data from the US Census Bureau's Census of State and Local Governments, which is conducted every 5 years and includes expenditure data from all county and sub-county jurisdictions in the United States^30^ Because complete data for all US counties are available only every 5 years and our study period for outcome measurement spanned a continuous 3-year period, we linked expenditure data from the 2017 census year. While prior studies employ 1-3 year lags in measuring exposure and outcome to mitigate against reverse causality, this was not feasible with our data. The AHRF compiles key information on health care resources at the county level and also includes social and economic characteristics of counties from other Census data sources.

### Outcome variables

We examined 5 outcomes spanning annual primary care use and avoidable hospital utilization: (1) primary care visits, (2) ED visits, (3) all-cause ambulatory care-sensitive condition (ACSC) admissions (ie, preventable hospitalizations), (4) acute condition ACSC admissions, and (5) chronic condition ACSC admissions. We identified visits to primary care clinicians using claims for evaluation and management visits with Current Procedural Terminology codes, consistent with prior methods.^31^ We measured ED visits for any cause using the Yale ED visit definition, which uses the carrier, inpatient, and outpatient files.^32^ For measures of preventable inpatient utilization, we measured ACSC admissions using the Prevention Quality Indicators specifications developed by the Agency for Healthcare Quality and Research.^33^ Acute condition ACSC admissions included diagnoses such as bacterial pneumonia or urinary tract infection, while chronic condition ACSC admissions included diagnoses such as heart failure or diabetes.

### Exposure variables

We examined the following types of social spending as the exposures of interest: welfare, public transit, housing and community development, and other core social services. The latter category encompassed line-item expenditures on building inspections, fire and first responders, utilities, and waste and sewer management. We selected these types of social spending based on prior work identifying health-related social needs associated with health care utilization among Medicare populations.^34-36^ We used direct expenditures, which measure operational costs and reflect service provision, and excluded capital outlays, which capture construction and purchasing expenditures and may not indicate changes in policy or programming. We standardized spending amounts as per capita measures using the county's population size.^11^ Given their non-normal distribution, we categorized spending as quintiles with the exception of public transit spending. Because a large number of beneficiaries (>100 000) lived in counties with zero spending on public transit, we categorized nonzero spending counties into quartiles.

### Adjustment variables

We adjusted for multiple observable covariates at both the beneficiary and county levels that may confound the relationship between governmental social spending and health care use. These included beneficiary age, sex, race, and ethnicity, and 21 clinical comorbidities using the Chronic Conditions Warehouse taxonomy.^37^ Race and ethnicity categories were based on the Research Triangle Institute race code algorithm and include Asian/Pacific Islander, American Indian/Alaska Native, non-Hispanic Black, Hispanic, Other, Unknown, and non-Hispanic White. Ideally, Asian and Pacific Islander beneficiary data would be disaggregated, but this is currently unavailable in Medicare data. Observations with unknown or missing race were excluded. Prior work has demonstrated that patients from minoritized backgrounds experience higher rates of ACSC admissions.^38-42^ Because individual differences in eligibility or need for social services may also confound the relationship between social spending and outcomes, we adjusted for the beneficiary social deprivation index (SDI), which is a composite measure of neighborhood-level social disadvantage used in prior studies to examine measures of health care access, quality of care, and health outcomes.^43-49^

We included county median household income and unemployment rate as measures of the strength of the local economy, primary care providers per capita and hospital beds per capita as measures of health care supply factors, and the Herfindahl–Hirschman Index (HHI) to capture hospital market concentration. The HHI was calculated using hospital admissions and county share of admissions from the American Hospital Association Annual Survey data.^50^ We then categorized counties as unconcentrated (HHI < 1500), moderately concentrated (HHI 1500-2499), highly concentrated (HHI 2500-4999), and very highly concentrated (HHI > 5000). We accounted for rural–urban status, as rural communities have less financial resources and less population density, which may influence the reach or uptake of social and health services. We defined rural–urban status based on residential zip code rural–urban commuting codes for the most recent year available.^51-55^ To account for geographic differences in purchasing power for goods, housing, utilities, and other services, we also included county-level regional price parities from the US Department of Commerce.^56^ Finally, to account for differences in political beliefs regarding the role of government, we used 2016 county presidential election returns from the MIT Election Data and Science Lab (https://www.healthaffairs.org/doi/full/10.1377/hlthaff.2022.00085). Because the state of Alaska reports elections at the congressional district level and not the county, we excluded beneficiaries living in Alaska.

### Statistical analysis

We examined the association between governmental spending categories and each outcome by fitting beneficiary-level multivariable Poisson regression models, adjusting for beneficiary and county characteristics. We examined each category of social spending separately due to concerns of collider bias^57^ and difficulty in interpreting the results when controlling for other forms of social spending simultaneously. We included the number of years a beneficiary was in the study as an offset term. Standard errors were clustered at the county level due to the hierarchical nature of the data and because the exposure is measured at the county level.^58^ While we accounted for a broad set of county-level covariates, there are likely regional differences with respect to state policy environments and administrative differences with local governments that influence local decisions on social spending. For example, states may differ in how they distribute state funding to local governments and the degree to which state governments override or hold jurisdiction over certain policy issues. Additionally, states may vary with regards to utilization patterns for Medicare beneficiaries, especially as some states may have more low-income older adults enrolled in managed care plans. To account for these sources of omitted variable bias, we used state fixed effects, which effectively compare outcomes of beneficiaries within the same state.

We report results as adjusted rate ratios and adjusted mean differences for each outcome, comparing patients living in higher spending counties with those in the lowest-spending counties (in the case of public transit, those in zero-spending counties were the referent group). To calculate adjusted mean differences (eg, average marginal effects), we used the marginal standardization technique to estimate the differences in predicted outcomes for each quintile or quartile for each patient and averaged over the distribution of covariates in our data.^59^ We rescaled the adjusted differences in rates to per 1000 beneficiaries per year for ease of interpretation. Because of multiple comparisons, we used the Benjamini–Hochberg procedure to control the false discovery rate, using an adjusted *P*-value <0.05 for statistical significance.^60^ This approach is less conservative compared to other procedures that control the familywise error rate, such as the Bonferroni procedure.

All analyses were conducted with Stata 16.0 MP. The institutional review board at the University of California, Los Angeles, deemed this study exempt from full approval and waived informed consent.

### Sensitivity analyses

We conducted several sensitivity analyses to test the robustness of our findings. First, because social spending has benefits that may accrue disproportionately to lower income individuals or those with greater need, we recalculated social spending variables in our models using dollars per number of people in a county living under the federal poverty line. Second, we examined social spending as a percentage of total spending. Third, we treated spending as a continuous variable, log-transforming spending per capita. Fourth, we substituted median household income with the county poverty rate in our models because poverty rates may serve as stronger measures of need for spending and county economic resources. Finally, because the effects of social spending types may flow through other programs, we controlled for all other social spending simultaneously.

## Results

The final sample consisted of 605 732 dual-eligible Medicare beneficiaries over the age of 65 residing in 3094 counties or county equivalents. Notably, dual-eligible beneficiaries lived primarily in urban counties (80.3%), were racially and ethnically diverse (17.1% Hispanic, 15.3% Black, and 10.4% Asian or Pacific Islander), and lived in relatively more disadvantaged areas (mean SDI 59.7, Table 1). Median spending per capita for categories of social services ranged from $0 per capita for public transit to $485.17 on other social services across counties (Table 2). Welfare spending was correlated with public transit and other services (eTable S1).

**Table 1.** Characteristics of low-income Medicare beneficiaries, 20% random sample of fee-for-service beneficiaries.

Patient characteristics	%
No. of beneficiaries	605 732
Age, mean (sd)	78.1 (9.0)
Female	400 329 (66.0%)
Race	
White	334 613 (55.2%)
Hispanic	103 503 (17.1%)
Black	92 637 (15.3%)
Asian/Pacific Islander	62 920 (10.4%)
American Indian/Alaska Native	6126 (1.0%)
Other	5933 (1.0%)
SDI, mean (sd)	59.7 (27.6)
Urban	481 022 (79.4%)
*Chronic conditions*	
Hypertension	460 450 (76.0%)
Hyperlipidemia	324 927 (53.6%)
Rheumatoid arthritis or osteoarthritis	278 174 (45.9%)
Diabetes	269 723 (44.5%)
Chronic kidney disease	246 660 (40.7%)
Anemia	245 549 (40.5%)
Ischemic heart disease	243 980 (40.3%)
Depression	197 194 (32.6%)
Congestive heart failure	180 819 (29.9%)
Chronic obstructive pulmonary disease	136 620 (22.6%)
Hypothyroid	124 124 (20.5%)
Cataracts	103 322 (17.1%)
Atrial fibrillation	70 765 (11.7%)
Osteoporosis	68 203 (11.3%)
Glaucoma	60 774 (10.0%)
Benign prostatic hyperplasia	54 734 (9.0%)
Stroke/transient ischemic attack	50 412 (8.3%)
Cancer (all types)	49 598 (8.2%)
Asthma	41 316 (6.8%)
Hip fracture	9932 (1.6%)
Acute myocardial infarction	8941 (1.5%)
*Outcomes*	*Mean* (*sd*)
Number of annual primary care visits (per beneficiary per year)	3.69 (5.19)
All-cause ED visits (per beneficiary per year)	2.00 (3.48)
Preventable hospitalizations (per beneficiary per year)	0.15 (0.51)
Preventable hospitalizations from acute conditions (per beneficiary per year)	0.05 (0.21)
Preventable hospitalizations from chronic conditions (per beneficiary per year)	0.10 (0.44)

**Table 2.** Characteristics of counties in the sample.

County characteristics	Median (IQR) or %
Number of counties	3094
Population size	26 244 (11 301-68 341)
Unemployment rate, %	4.4 (3.5-5.3)
Poverty rate, %	14.2 (10.7-18.5)
Median household income, $	51 676 (44 129-59 587)
Regional price parity, %	88.1 (85.6-92.3)
Primary care providers (per 1000)	0.47 (0.29-0.69)
Hospital Beds (per 1000)	1.87 (0.71-3.60)
Market concentration	
Unconcentrated	22.5
Moderately concentrated	2.1
Highly concentrated	7.6
Very highly concentrated	67.8
Political leaning	
Democratic	3.9
Democratic-leaning	15.7
Republican-leaning	40.4
Republican	39.9
Total Gov expenditures per capita, $	2617.28 (1722.36-3870.15)
Welfare spending per capita, $	12.47 (1.90-120.34)
Public transit spending per capita, $	0.00 (0.00-6.93)
Housing and community development spending per capita), $	33.75 (4.52-86.67)
Other social services spending (utilities, waste/sewer, inspections), $	485.17 (304.59-798.88)
Public health spending per capita, $	53.19 (18.27-125.96)

For the outcome of primary care visits, we found that beneficiaries living in the highest quintile of housing and community development spending had a higher adjusted rate of primary care visits per beneficiary per year relative to those in the lowest quintile (adjusted IRR 1.11; 95% CI, 1.04-1.19; adjusted *P* = 0.02; Table 3). We did not observe a significant relationship between other types of social spending and primary care visits in our primary analysis. Additionally, we did not observe any significant relationships between any type of social spending and ED visits.

**Table 3.** Adjusted associations between social spending type and utilization of primary care and the ED.

	Primary care visits					ED visits				
Spending category	IRR	95% CI	AME	95% CI	Adj. *P*^a^	IRR	95% CI	AME	95% CI	Adj. *P*^a^
Welfare (ref = Quintile 1)										
Quintile 2	1.02	0.97-1.08	+162.1	(−255.3 to +579.5)	0.94	1.00	0.97-1.02	−6.8	(−108.1 to +94.4)	0.99
Quintile 3	1.08	1.02-1.15	+608.9	(+128.2 to +1089.6)	0.26	0.97	0.94-1.00	−113.3	(−227.0 to +0.4)	0.48
Quintile 4	1.08	0.99-1.17	+568.5	(−76.8 to +1213.8)	0.43	0.99	0.96-1.02	−53.8	(−174.7 to +67.0)	0.95
Quintile 5	1.09	0.99-1.19	+655.8	(−51.7 to +1363.4)	0.43	0.98	0.93-1.03	−73.6	(−273.2 to +12.6)	0.94
Public transit (ref = zero-spending counties)										
Quartile 1	1.08	1.00-1.17	+642.4	(−28.6.7 to +1313.3)	0.11	1.03	1.01-1.06	+129.7	(+20.5 to +238.8)	0.05
Quartile 2	1.07	1.03-1.12	+603.0	(+221.2 to +984.7)	0.03	1.02	0.99-1.04	+71.3	(−25.2 to +167.8)	0.24
Quartile 3	1.00	0.95-1.06	+7.4	(−444.6 to +459.5)	0.97	1.03	1.01-1.06	+134.1	(+24.8 to +243.4)	0.05
Quartile 4	0.98	0.93-1.03	−168.4	(−575.8 to +238.9)	0.56	1.00	0.97-1.03	+8.2	(−109.6 to +125.9)	0.96
										
Housing (ref = Quintile 1)										
Quintile 2	1.03	0.97-1.10	+226.1	(−251.0 to +703.1)	0.54	1.02	0.99-1.05	+75.7	(−41.1 to +192.5)	0.45
Quintile 3	1.15	1.07-1.23	+1066.6	(+525.5 to +1607.7)	<0.01	1.00	0.98-1.03	+8.3	(−97.0 to +113.7)	0.92
Quintile 4	1.11	1.04-1.18	+819.3	(+344.7 to +1293.9)	0.01	1.00	0.97-1.03	+3.9	(−109.5 to +117.3)	0.95
Quintile 5	1.11	1.04-1.19	+803.6	(+283.3 to +1323.9)	0.02	0.97	0.94-1.01	−104.5	(−235.3 to +26.3)	0.33
Other social services (ref = Quintile 1)										
Quintile 2	1.07	0.99-1.15	+509.7	(−90.0 to +1109.3)	0.45	0.99	0.97-1.02	−31.7	(−130.2 to +66.8)	0.96
Quintile 3	1.05	1.00-1.11	+407.4	(+19.4 to +795.4)	0.65	1.02	1.00-1.05	+84.8	(−13.0 to +182.6)	0.45
Quintile 4	1.01	0.95-1.07	+93.2	(−363.8 to +550.2)	0.92	1.00	0.97-1.03	−1.9	(−122.6 to +118.8)	0.98
Quintile 5	1.06	1.00-1.12	+440.8	(−24.1 to +905.6)	0.60	1.00	0.97-1.03	+9.6	(−110.1 to +129.4)	1.00
Observations	605 732					605 732				

Results presented for each category of social spending and outcome are derived from separate regression models. Models adjust for beneficiary-level characteristics (age, sex, race, zip code SDI, urbanicity, and comorbidities), county-level characteristics (median household income, unemployment rate, PCP density, hospital beds, public health spending, market concentration, purchasing power, and party vote share), and include state fixed effects.

Abbreviations: AME, average marginal effects per 1000 beneficiaries per year; CI, confidence interval;IRR, incidence rate ratio.

^a^Adjusted for multiple comparisons using the Benjamini-Hochberg step up procedure.

Relative to beneficiaries living in counties that had zero spending on public transit, those in the highest quartile had a lower rate of preventable hospitalizations (IRR 0.94; 95% CI, 0.90-0.98; adjusted *P* = 0.03; Table 4) and a lower rate of preventable hospitalizations from acute conditions (IRR 0.92; 95% CI, 0.87-0.97; adjusted *P* = 0.03; Table 4). We also found that living in the highest quintile for housing and community development spending was associated with lower preventable hospitalizations (IRR 0.93; 95% CI, 0.88-0.98; adjusted *P* = 0.02; Table 4) and lower preventable hospitalizations from acute conditions (IRR 0.90; 95% CI, 0.83-0.97; adjusted *P* = 0.02; Table 4) per beneficiary per year. There was no evidence that adjusted rates of chronic condition hospitalizations differed between high- and low-spending counties for any category of social services.

**Table 4.** Adjusted associations between social spending type and preventable hospitalizations (overall ambulatory care-sensitive condition hospitalizations and acute and chronic types).

	Preventable hospitalizations, overall (PQI90)					Acute condition preventable hospitalizations (PQI91)					Chronic condition preventable hospitalizations (PQI92)				
Spending category	IRR	95% CI	AME	95% CI	Adj. *P*^a^	IRR	95% CI	AME	95% CI	Adj. *P*^a^	IRR	95% CI	AME	95% CI	Adj. *P*^a^
Welfare (ref = Quintile 1)															
Quintile 2	1.00	0.95-1.05	0.0	(−13.3 to +13.1)	0.99	1.00	0.94-1.07	+0.3	(−5.3 to +5.9)	0.97	1.00	0.95-1.06	+0.5	(−9.5 to +10.4)	0.97
Quintile 3	1.00	0.95-1.05	−0.7	(−13.7 to+12.2)	0.91	0.97	0.91-1.03	−2.8	(−8.6 to +3.0)	0.99	1.02	0.96-1.07	+3.0	(−6.8 to +12.8)	0.92
Quintile 4	1.00	0.95-1.05	−1.0	(−15.2 to +13.1)	0.88	0.99	0.92-1.06	−1.2	(−7.6 to +5.2)	0.99	1.01	0.95-1.06	+1.0	(−9.6 to +11.6)	0.99
Quintile 5	1.03	0.97-1.09	+8.0	(−8.7 to +24.6)	0.95	1.05	0.96-1.14	+4.2	(−4.0 to +12.4)	0.99	1.02	0.96-1.09	+4.1	(−8.3 to +16.4)	0.92
Public transit (ref = zero-spending counties)															
Quartile 1	0.99	0.95-1.03	−1.8	(−13.3 to +9.7)	0.89	0.98	0.92-1.04	−1.9	(−7.7 to +3.9)	0.65	1.00	0.96-1.05	+0.5	(−8.5 to +9.5)	0.96
Quartile 2	0.97	0.93-1.01	−8.5	(−19.8 to +2.8)	0.24	0.97	0.92-1.03	−2.6	(−7.9 to +2.8)	0.49	0.97	0.93-1.02	−5.2	(−13.6 to +3.1)	0.34
Quartile 3	0.95	0.91-0.99	−14.2	(−25.3 to −3.1)	0.05	0.92	0.87-0.98	−7.4	(−13.1 to −1.8)	0.05	0.97	0.93-1.01	−6.4	(−14.7 to +1.9)	0.26
Quartile 4	0.94	0.90-0.98	−16.9	(−28.5 to −5.4)	0.03	0.92	0.87-0.97	−7.6	(−12.8 to −2.4)	0.03	0.95	0.91-1.00	−9.0	(−17.7 to −0.3)	0.11
Housing (ref = Quintile 1)															
Quintile 2	0.99	0.95-1.04	−1.8	(−15.6 to +12.0)	0.94	0.98	0.92-1.05	−1.9	(−8.4 to +4.6)	0.71	1.01	0.95-1.06	+1.0	(−9.7 to +11.7)	0.92
Quintile 3	0.97	0.92-1.02	−8.6	(−22.7 to +5.4)	0.40	0.96	0.89-1.03	−4.2	(−10.9 to +2.6)	0.41	0.98	0.93-1.04	−3.5	(−14.4 to +7.4)	0.70
Quintile 4	0.97	0.92-1.01	−9.5	(−22.7 to +3.7)	0.39	0.96	0.90-1.03	−3.7	(−10.0 to +2.5)	0.40	0.98	0.92-1.03	−4.7	(−15.0 to +5.7)	0.54
Quintile 5	0.93	0.88-0.98	−20.4	(−35.0 to −5.9)	0.02	0.90	0.83-0.97	−10.0	(−17.1 to −3.0)	0.02	0.95	0.90-1.01	−8.9	(−20.0 to +2.2)	0.33
Other social services (ref = Quintile 1)															
Quintile 2	1.01	0.97-1.06	+3.4	(−8.4 to +15.2)	0.95	1.00	0.94-1.07	+0.4	(−5.4 to +6.2)	0.96	1.02	0.97-1.07	+3.1	(−6.1 to +12.2)	0.96
Quintile 3	1.02	0.98-1.06	+6.2	(−5.4 to +17.7)	0.85	1.00	0.94-1.06	−0.3	(−5.9 to +5.3)	0.96	1.04	0.99-1.09	+6.9	(−2.0 to +15.7)	0.52
Quintile 4	0.99	0.95-1.03	−3.0	(−14.8 to +8.8)	0.91	0.98	0.92-1.04	−2.3	(−8.1 to +3.5)	0.96	1.00	0.95-1.04	−0.9	(−9.8 to +7.9)	1.00
Quintile 5	0.98	0.94-1.03	−5.6	(−18.5 to +7.3)	0.96	0.97	0.91-1.03	−3.2	(−9.1 to +2.7)	0.85	0.99	0.93-1.04	−2.4	(−12.6 to +7.7)	0.91
Observations	605 732					605 732					605 732				

Results presented for each category of social spending and outcome are derived from separate regression models. Models adjust for beneficiary-level characteristics (age, sex, race, zip code SDI, urbanicity, and comorbidities), county-level characteristics (median household income, unemployment rate, PCP density, hospital beds, public health spending, market concentration, purchasing power, and party vote share), and include state fixed effects.

Abbreviations: AME, average marginal effects; CI, confidence interval; IRR, incidence rate ratio; PQI90—Prevention Quality Indicator 90, overall preventable hospitalizations; PQI91—Prevention Quality Indicator 91, preventable hospitalizations from acute conditions; PQI92—Prevention Quality Indicator 92, preventable hospitalizations from chronic conditions.

^a^Adjusted for multiple comparisons using the Benjamini–Hochberg step up procedure.

### Sensitivity analyses

Sensitivity analyses that recalculated social spending variables and used alternative adjustment variables yielded similar results as the main analysis (eTables S2-S6).

## Discussion

This national cross-sectional study of dual-eligible Medicare fee-for-service beneficiaries observed multiple associations between higher spending on 2 types of social services and health care utilization. We observed the strongest associations for spending on housing and community development. Higher rates of primary care visits among dual-eligible beneficiaries were associated with residence in the highest-spending counties for housing and community development, but no other form of social spending. Additionally, beneficiaries living in counties with higher spending on public transit and housing/community development experienced lower rates of preventable hospitalizations, with a clear dose–response relationship. These relationships were strongest for preventable hospitalizations for acute conditions. Notably, the association for public transit and utilization was significant for preventable hospitalizations, but not primary care visits.

Taken together, these findings suggest that local governments’ spending on housing is positively correlated with greater access to primary care, while spending on housing and public transit may be associated with more efficient use of acute care services in the form of lower preventable hospitalizations. Although existing ecological studies have shown a positive relationship between county expenditures on social services and population health outcomes, evidence is limited on whether greater social spending is associated with health care utilization at the person level. For example, 1 existing ecological study using county-level hospitalization rates from 32 states found that social spending was associated with lower preventable hospitalizations.^61^ However, ecological relationships do not necessarily translate into person-level relationships, a statistical phenomenon widely known as ecological fallacy; therefore, it remains unclear whether higher social spending leads to improved health care utilization at the person level. The current study addresses this knowledge gap by using person-level data, with adjustment for a large set of person-level demographics, SDI, and comorbidities, which more adequately captures the need for social services. Moreover, this study focuses on a national sample of low-income older adults, whose health care use may be specifically sensitive to variation in community social services.

This study also extends the literature by examining primary care visits, an important conceptual link between social spending and downstream use of acute care services. While we only observed a significant association with housing and community development spending, the use of state fixed effects in our analyses may have limited statistical power. Low-income patients experience greater unmet social needs, such as inadequate transportation, which predicts no-show rates in primary care.^62,63^ Thus, greater social spending may be linked to higher primary care visit rates, potentially through addressing barriers and unmet social needs. Greater access to primary care, in turn, is associated with lower use of acute care services for low-income adults generally and lower preventable hospitalizations for Medicare beneficiaries, specifically.^29,64^

We found that the associations were strongest and most consistent for public transit and housing with preventable hospitalizations. There are multiple reasons why public transit spending is associated with lower preventable hospitalizations. While this analysis was unable to examine causal mechanisms, older adults who experience financial strain disproportionately use public transit, especially in accessing medical care.^26^ Robustly funded public transit may help older adults adapt to aging-related mobility challenges and mitigate social isolation by accommodating assistive devices or wheelchairs.^65^ Low-income Medicare beneficiaries with worse transportation access experience delayed medical care, missed appointments, difficulty accessing pharmacies, medication non-adherence, and worsening of chronic conditions, which could lead to preventable hospitalizations.^66,67^ A recent study of low-income patients found that expansion of public transit decreased no-show appointments.^68^ Thus, public transit may facilitate outpatient care and early clinical interventions that could prevent the worsening of a condition that would lead to a hospitalization.

Housing and community development spending had the strongest relationship with primary care use and preventable hospitalizations. Living in the highest-spending counties was associated with a mean difference of 20 fewer preventable hospitalizations per 1000 beneficiaries. These findings underscore the relationship between housing affordability and access to health care for older adults. One in 3 older Medicare beneficiaries experience housing insecurity, with much higher rates among those with geriatric syndromes, such as frailty.^69^ Low-income older adults are more likely to be renters, and housing costs comprise a disproportionately larger portion of monthly budgets.^25^

We observed that social spending categories were consistently associated with acute condition preventable hospitalizations and not chronic ones, regardless of the type of social spending. One potential reason for this finding is that social services may be a determinant of timely access to primary care, but might not translate into continuity of care, which is needed for chronic disease management.^70^ Future studies, employing stronger causal designs or exploiting natural experiments, should examine the specific pathways between social spending, policy implementation, and health care utilization for individuals with high social and medical needs.

This work has implications for a variety of stakeholders. Our findings suggest that local social services and policies potentially complement the core function of the health care system in delivering medical care. Greater allocation of local funding on social policies, such as public transit, housing, and community development, may be associated with improvements in the health-related social needs of vulnerable older adults in Medicare, which could be the path through which social spending is associated with access to primary care and lower preventable hospitalizations. However, because the care of dual-eligible beneficiaries is concentrated in a small number of outpatient practices, investing in primary care is likely needed.^71^ These findings also have implications for post-acute care spending, given that hospitalizations may accelerate frailty and contribute to long-term care use, which comprises a persistently large share of spending in Medicare.^72^

### Limitations

This study has limitations. First, as an observational cross-sectional study, there is the potential for unobserved confounding, and the findings should not be interpreted as causal. Nonetheless, this study is a major contribution to the literature and includes a robust set of adjustment variables, including state fixed effects. Second, governmental expenditures are not a direct measure of the number or type of social services, the reach of services, or their effectiveness. Therefore, these results are similar to an intent-to-treat approach and potentially underestimate the true association of social spending with primary care visits and preventable hospitalizations. Third, eligibility for Medicaid differs by state, as some have higher and lower income criteria. However, income eligibility for older adults in Medicare is largely determined by the federal government.^73^ Additionally, our analyses employed state fixed effects, effectively comparing beneficiaries within the same state, which could limit statistical power. Fourth, this analysis excluded patients enrolled in Medicare Advantage plans, who may have different utilization patterns compared to those enrolled in fee-for-service. Fifth, these findings may not be generalizable outside of the dual-eligible Medicare population.

## Conclusion

Among low-income Medicare beneficiaries aged 65 years and older, those living in communities with greater governmental spending on housing and community development had higher primary care use. Those living in communities that spent more on housing and public transit experienced lower rates of preventable hospitalizations, particularly those from acute conditions. Governmental investments that address health-related social needs of low-income older adults may be an important factor in efforts to address primary care access and utilization of costly acute care services.

## Supplementary Material

qxae181_Supplementary_Data
