# Supplementary material for: Association between governmental spending on social services and health care use among low-income older adults
Source: Health Aff Sch. 2025 Jan 10;3(1):qxae181. doi: 10.1093/haschl/qxae181 (PMC11770339; doi:10.1093/haschl/qxae181)
Supplement: qxae181_Supplementary_Data [file qxae181_supplementary_data.zip › HAS_socspend_manuscript_appendix_revised.docx]

# **APPENDIX**

**eTable 1.** Correlation Matrix of Social Spending Variables and County Characteristics.

|  | Welfare | | Housing | | Public Transit | | Other services | | Public Health | | Median HHI | | Poverty rate | | Unemp. rate | | Beds per capita | | PCPs per capita | | Pop. Size | | RPP | | Mkt Conc. | | Dem Share |  |
| --- | --- | --- | --- | --- | --- | --- | --- | --- | --- | --- | --- | --- | --- | --- | --- | --- | --- | --- | --- | --- | --- | --- | --- | --- | --- | --- | --- | --- |
| Welfare | 1.00 |  | |  | |  | |  | |  | |  | |  | |  | |  | |  | |  | |  | |  | |  |
| Housing | 0.01 | 1.00 | |  | |  | |  | |  | |  | |  | |  | |  | |  | |  | |  | |  | |  |
| Public Transit | 0.28^***^ | 0.01 | | 1.00 | |  | |  | |  | |  | |  | |  | |  | |  | |  | |  | |  | |  |
| Other services | 0.15^***^ | 0.00 | | 0.03 | | 1.00 | |  | |  | |  | |  | |  | |  | |  | |  | |  | |  | |  |
| Public Health | 0.10^***^ | -0.01 | | 0.08^***^ | | 0.04^*^ | | 1.00 | |  | |  | |  | |  | |  | |  | |  | |  | |  | |  |
| Median HHI | 0.09^***^ | 0.01 | | 0.15^***^ | | 0.04^*^ | | 0.03 | | 1.00 | |  | |  | |  | |  | |  | |  | |  | |  | |  |
| Poverty rate | -0.09^***^ | -0.02 | | -0.04^*^ | | -0.06^***^ | | -0.03 | | -0.74^***^ | | 1.00 | |  | |  | |  | |  | |  | |  | |  | |  |
| Unemp. rate | -0.00 | -0.03 | | -0.01 | | -0.06^***^ | | 0.05^**^ | | -0.45^***^ | | 0.57^***^ | | 1.00 | |  | |  | |  | |  | |  | |  | |  |
| Beds per capita | 0.03 | 0.02 | | 0.02 | | 0.10^***^ | | 0.02 | | -0.08^***^ | | 0.03 | | -0.06^***^ | | 1.00 | |  | |  | |  | |  | |  | |  |
| PCPs per capita | 0.09^***^ | -0.01 | | 0.14^***^ | | 0.11^***^ | | 0.05^*^ | | 0.30^***^ | | -0.19^***^ | | -0.17^***^ | | 0.24^***^ | | 1.00 | |  | |  | |  | |  | |  |
| Pop. Size | 0.10^***^ | -0.00 | | 0.25^***^ | | 0.02 | | 0.04^*^ | | 0.26^***^ | | -0.08^***^ | | -0.03 | | -0.01 | | 0.19^***^ | | 1.00 | |  | |  | |  | |  |
| RPP | 0.14^***^ | -0.00 | | 0.25^***^ | | 0.04^*^ | | 0.11^***^ | | 0.71^***^ | | -0.40^***^ | | -0.17^***^ | | -0.08^***^ | | 0.36^***^ | | 0.44^***^ | | 1.00 | |  | |  | |  |
| Mkt Conc. | -0.03 | -0.01 | | -0.06^**^ | | 0.05^**^ | | -0.02 | | -0.06^***^ | | 0.00 | | -0.04^*^ | | 0.23^***^ | | 0.16^***^ | | -0.18^***^ | | -0.14^***^ | | 1.00 | |  | |  |
| Dem Share | 0.14^***^ | 0.01 | | 0.22^***^ | | 0.02 | | 0.08^***^ | | 0.16^***^ | | 0.18^***^ | | 0.21^***^ | | -0.02 | | 0.30^***^ | | 0.33^***^ | | 0.45^***^ | | -0.10^***^ | | 1.00 | |  |
| Counties | 3,094 | |  | |  | |  | |  | |  | |  | |  | |  | |  | |  | |  | |  | |  |  |

^*^ p < 0.05, ^**^ p < 0.01, ^***^ p < <0.011

**Abbrev:** HHI, household income; unemp, unemployment; PCPs, primary care providers; Pop, population; RPP, regional purchasing power; Mkt Conc, market concentration; Dem Share, democratic voting share

**eTable 2.** Associations Between Social Spending (Recalculated as Per Person in Poverty) and Primary Care Visits, ED Visits, and ACSC Hospitalizations.

|  | **Primary Care Visits** | | | **ED Visits** | | | **PQI90** | | | **PQI91** | | | **PQI92** | | | |
| --- | --- | --- | --- | --- | --- | --- | --- | --- | --- | --- | --- | --- | --- | --- | --- | --- |
| Spending | IRR | 95% CI | p | IRR | 95% CI | p | IRR | 95% CI | p | IRR | 95% CI | p | IRR | 95% CI | p | |
| **Welfare (ref = Quintile 1)** |  |  |  |  |  |  |  |  |  |  |  |  |  |  |  | |
| Quintile 2 | 1.04 | 0.98 - 1.10 | 0.23 | 0.99 | 0.97 - 1.02 | 0.61 | 1.00 | 0.95 - 1.05 | 0.96 | 1.00 | 0.94 - 1.06 | 0.96 | 1.00 | 0.95 - 1.06 | 0.91 | |
| Quintile 3 | 1.08 | 1.00 - 1.17 | 0.05 | 0.98 | 0.95 - 1.01 | 0.19 | 1.01 | 0.96 - 1.06 | 0.75 | 0.99 | 0.93 - 1.06 | 0.77 | 1.02 | 0.96 - 1.08 | 0.52 | |
| Quintile 4 | 1.05 | 0.98 - 1.12 | 0.16 | 0.99 | 0.96 - 1.02 | 0.45 | 1.00 | 0.95 - 1.05 | 0.97 | 1.01 | 0.94 - 1.08 | 0.85 | 1.00 | 0.94 - 1.06 | 0.91 | |
| Quintile 5 | 1.02 | 0.94 - 1.10 | 0.68 | 0.98 | 0.94 - 1.03 | 0.46 | 1.04 | 0.97 - 1.11 | 0.27 | 1.07 | 0.97 - 1.18 | 0.15 | 1.02 | 0.95 - 1.09 | 0.66 | |
|  |  |  |  |  |  |  |  |  |  |  |  |  |  |  |  | |
| **Public Transit (ref = zero spending)** |  |  |  |  |  |  |  |  |  |  |  |  |  |  |  | |
| Quartile 1 | 1.09 | 1.01 - 1.18 | 0.03 | 1.04 | 1.01 - 1.07 | 0.01 | 1.00 | 0.96 - 1.05 | 0.82 | 1.00 | 0.94 - 1.06 | 0.99 | 1.01 | 0.96 - 1.06 | 0.70 | |
| Quartile 2 | 1.08 | 1.02 - 1.14 | 0.01 | 1.01 | 0.98 - 1.04 | 0.50 | 0.96 | 0.92 - 1.00 | 0.05 | 0.94 | 0.88 - 1.00 | 0.06 | 0.98 | 0.93 - 1.02 | 0.27 | |
| Quartile 3 | 0.97 | 0.93 - 1.02 | 0.22 | 1.02 | 1.00 - 1.05 | 0.09 | 0.94 | 0.90 - 0.97 | <0.01 | 0.91 | 0.86 - 0.97 | <0.01 | 0.95 | 0.91 - 0.99 | 0.02 | |
| Quartile 4 | 0.99 | 0.94 - 1.04 | 0.68 | 1.01 | 0.98 - 1.04 | 0.48 | 0.95 | 0.91 - 0.99 | 0.03 | 0.94 | 0.89 - 1.00 | 0.04 | 0.96 | 0.91 - 1.01 | 0.09 | |
|  |  |  |  |  |  |  |  |  |  |  |  |  |  |  |  | |
| **Housing (ref = Quintile 1)** |  |  |  |  |  |  |  |  |  |  |  |  |  |  |  | |
| Quintile 2 | 1.10 | 1.02 - 1.18 | 0.01 | 1.01 | 0.98 - 1.04 | 0.60 | 0.97 | 0.93 - 1.02 | 0.25 | 0.96 | 0.90 - 1.03 | 0.23 | 0.98 | 0.93 - 1.04 | 0.56 | |
| Quintile 3 | 1.15 | 1.07 - 1.25 | <0.01 | 1.00 | 0.97 - 1.03 | 0.98 | 0.97 | 0.92 - 1.01 | 0.15 | 0.96 | 0.90 - 1.03 | 0.24 | 0.97 | 0.92 - 1.03 | 0.35 | |
| Quintile 4 | 1.14 | 1.06 - 1.22 | <0.01 | 1.00 | 0.97 - 1.03 | 0.91 | 0.95 | 0.91 - 1.00 | 0.05 | 0.93 | 0.87 - 1.00 | 0.04 | 0.97 | 0.92 - 1.02 | 0.27 | |
| Quintile 5 | 1.17 | 1.09 - 1.26 | <0.01 | 0.96 | 0.92 - 0.99 | 0.02 | 0.92 | 0.87 - 0.97 | <0.01 | 0.89 | 0.83 - 0.96 | <0.01 | 0.94 | 0.89 - 1.00 | 0.04 | |
|  |  |  |  |  |  |  |  |  |  |  |  |  |  |  |  | |
| **Other social services (ref = Quintile 1)** |  |  |  |  |  |  |  |  |  |  |  |  |  |  |  | |
| Quintile 2 | 0.99 | 0.89 - 1.10 | 0.82 | 0.99 | 0.97 - 1.02 | 0.64 | 0.97 | 0.93 - 1.01 | 0.13 | 0.96 | 0.90 - 1.03 | 0.24 | 0.97 | 0.93 - 1.02 | 0.24 | |
| Quintile 3 | 1.00 | 0.91 - 1.10 | 0.97 | 1.03* | 1.01 - 1.06 | 0.02 | 1.01 | 0.97 - 1.06 | 0.63 | 1.00 | 0.94 - 1.06 | 0.98 | 1.01 | 0.96 - 1.06 | 0.63 | |
| Quintile 4 | 0.97 | 0.88 - 1.06 | 0.49 | 1.02 | 0.99 - 1.06 | 0.12 | 0.98 | 0.94 - 1.03 | 0.41 | 0.96 | 0.90 - 1.03 | 0.25 | 0.98 | 0.93 - 1.04 | 0.56 | |
| Quintile 5 | 0.99 | 0.90 - 1.09 | 0.84 | 0.98 | 0.94 - 1.02 | 0.38 | 0.97 | 0.92 - 1.02 | 0.24 | 0.96 | 0.89 - 1.04 | 0.34 | 0.97 | 0.91 - 1.03 | 0.31 | |
|  |  |  |  |  |  |  |  |  |  |  |  |  |  |  |  | |
| Observations | 605,732 | |  | 605,732 | |  | 605,732 | |  | 605,732 | |  | 605,732 | |  | |
| **Abbrev**: IRR, incidence rate ratio; CI, confidence interval.  **Note:** Models adjust for beneficiary-level characteristics (age, sex, race, zip-code SDI, comorbidities), county-level characteristics (median household income, unemployment rate, PCP density, hospital beds, public health spending, market concentration, purchasing power, party vote share), and include state fixed effects. | | | | | | | | | | | | | | | |  |

**eTable 3.** Associations Between Social Spending (Recalculated as % of Total Expenditures) and Primary Care Visits, ED Visits, and ACSC Hospitalizations.

|  | **Primary Care Visits** | | | **ED Visits** | | | **PQI90** | | | **PQI91** | | | **PQI92** | | | |
| --- | --- | --- | --- | --- | --- | --- | --- | --- | --- | --- | --- | --- | --- | --- | --- | --- |
| Spending | IRR | 95% CI | p | IRR | 95% CI | p | IRR | 95% CI | p | IRR | 95% CI | p | IRR | 95% CI | p | |
| **Welfare (ref = Quintile 1)** |  |  |  |  |  |  |  |  |  |  |  |  |  |  |  | |
| Quintile 2 | 1.00 | 0.95 - 1.06 | 0.97 | 0.99 | 0.96 - 1.01 | 0.34 | 1.00 | 0.95 - 1.05 | 0.99 | 1.02 | 0.96 - 1.09 | 0.51 | 0.99 | 0.94 - 1.05 | 0.75 | |
| Quintile 3 | 1.05 | 0.99 - 1.11 | 0.13 | 0.99 | 0.96 - 1.01 | 0.32 | 1.00 | 0.96 - 1.05 | 0.89 | 0.99 | 0.93 - 1.06 | 0.83 | 1.01 | 0.96 - 1.07 | 0.63 | |
| Quintile 4 | 1.07 | 0.97 - 1.18 | 0.16 | 0.98 | 0.95 - 1.01 | 0.22 | 0.98 | 0.93 - 1.03 | 0.49 | 0.97 | 0.91 - 1.04 | 0.42 | 0.99 | 0.93 - 1.05 | 0.72 | |
| Quintile 5 | 1.14 | 1.03 - 1.27 | 0.01 | 0.97 | 0.92 - 1.01 | 0.14 | 1.00 | 0.94 - 1.06 | 1.00 | 1.00 | 0.91 - 1.09 | 0.92 | 1.00 | 0.94 - 1.07 | 0.92 | |
|  |  |  |  |  |  |  |  |  |  |  |  |  |  |  |  | |
| **Public Transit (ref = zero spending)** |  |  |  |  |  |  |  |  |  |  |  |  |  |  |  | |
| Quartile 1 | 1.04 | 0.99 - 1.09 | 0.09 | 1.04 | 1.01 - 1.07 | 0.02 | 1.00 | 0.96 - 1.04 | 0.92 | 1.00 | 0.94 - 1.06 | 0.97 | 1.00 | 0.95 - 1.05 | 0.95 | |
| Quartile 2 | 1.08 | 1.00 - 1.17 | 0.06 | 1.03 | 1.00 - 1.05 | 0.04 | 0.97 | 0.94 - 1.01 | 0.21 | 0.96 | 0.91 - 1.02 | 0.24 | 0.98 | 0.94 - 1.03 | 0.46 | |
| Quartile 3 | 0.99 | 0.93 - 1.05 | 0.68 | 1.03 | 1.00 - 1.07 | 0.03 | 0.95 | 0.91 - 0.99 | 0.01 | 0.91 | 0.86 - 0.97 | <0.01 | 0.97 | 0.93 - 1.01 | 0.15 | |
| Quartile 4 | 1.03 | 0.98 - 1.08 | 0.24 | 1.00 | 0.97 - 1.03 | 0.89 | 0.94 | 0.90 - 0.98 | <0.01 | 0.93 | 0.88 - 0.98 | 0.01 | 0.95 | 0.91 - 1.00 | 0.03 | |
|  |  |  |  |  |  |  |  |  |  |  |  |  |  |  |  | |
| **Housing (ref = Quintile 1)** |  |  |  |  |  |  |  |  |  |  |  |  |  |  |  | |
| Quintile 2 | 1.08 | 1.01 - 1.16 | 0.02 | 1.03 | 1.00 - 1.07 | 0.05 | 1.00 | 0.95 - 1.06 | 0.87 | 0.98 | 0.92 - 1.05 | 0.64 | 1.02 | 0.96 - 1.08 | 0.50 | |
| Quintile 3 | 1.12 | 1.05 - 1.19 | <0.01 | 1.00 | 0.98 - 1.03 | 0.73 | 0.96 | 0.92 - 1.01 | 0.15 | 0.95 | 0.89 - 1.02 | 0.13 | 0.98 | 0.92 - 1.04 | 0.49 | |
| Quintile 4 | 1.16 | 1.09 - 1.25 | <0.01 | 0.99 | 0.97 - 1.02 | 0.68 | 0.95 | 0.91 - 1.00 | 0.06 | 0.93 | 0.87 - 0.99 | 0.03 | 0.97 | 0.92 - 1.03 | 0.37 | |
| Quintile 5 | 1.17 | 1.09 - 1.26 | <0.01 | 0.97 | 0.94 - 1.00 | 0.09 | 0.93 | 0.88 - 0.98 | <0.01 | 0.89 | 0.83 - 0.95 | <0.01 | 0.96 | 0.91 - 1.02 | 0.16 | |
|  |  |  |  |  |  |  |  |  |  |  |  |  |  |  |  | |
| **Other social services (ref = Quintile 1)** |  |  |  |  |  |  |  |  |  |  |  |  |  |  |  | |
| Quintile 2 | 0.97 | 0.92 - 1.03 | 0.34 | 1.06 | 1.01 - 1.10 | 0.01 | 1.05 | 1.00 - 1.10 | 0.04 | 1.07 | 0.99 - 1.16 | 0.08 | 1.04 | 0.99 - 1.09 | 0.09 | |
| Quintile 3 | 1.00 | 0.94 - 1.06 | 0.97 | 1.07 | 1.03 - 1.12 | <0.01 | 1.05 | 1.00 - 1.10 | 0.03 | 1.07 | 0.99 - 1.14 | 0.07 | 1.04 | 0.99 - 1.09 | 0.10 | |
| Quintile 4 | 1.09 | 1.01 - 1.16 | 0.02 | 1.03 | 0.98 - 1.08 | 0.30 | 1.02 | 0.98 - 1.07 | 0.30 | 1.03 | 0.95 - 1.10 | 0.49 | 1.02 | 0.98 - 1.07 | 0.35 | |
| Quintile 5 | 1.03 | 0.96 - 1.10 | 0.39 | 1.05 | 1.01 - 1.09 | 0.02 | 1.01 | 0.96 - 1.06 | 0.75 | 1.00 | 0.93 - 1.07 | 0.98 | 1.01 | 0.96 - 1.07 | 0.64 | |
|  |  |  |  |  |  |  |  |  |  |  |  |  |  |  |  | |
| Observations | 605,732 | |  | 605,732 | |  | 605,732 | |  | 605,732 | |  | 605,732 | |  | |
| **Abbrev**: IRR, incidence rate ratio; CI, confidence interval.  **Note:** Models adjust for beneficiary-level characteristics (age, sex, race, zip-code SDI, comorbidities), county-level characteristics (median household income, unemployment rate, PCP density, hospital beds, public health spending, market concentration, purchasing power, party vote share), and include state fixed effects. | | | | | | | | | | | | | | | |  |

**eTable 4.** Associations Between Social Spending (log-transformed) and Primary Care Visits, ED Visits, and ACSC Hospitalizations.

|  | **Primary Care Visits** | | | | **ED Visits** | | | | | | **PQI90** | | | | | | **PQI91** | | | | | | **PQI92** | | | | | | |
| --- | --- | --- | --- | --- | --- | --- | --- | --- | --- | --- | --- | --- | --- | --- | --- | --- | --- | --- | --- | --- | --- | --- | --- | --- | --- | --- | --- | --- | --- |
| Spending | IRR | 95% CI | p | | IRR | | 95% CI | | p | | IRR | | 95% CI | | p | | IRR | | 95% CI | | p | | IRR | | 95% CI | | p | | |
| **Welfare** | 1.00 | 1.00 - 1.01 | 0.03 | | 1.00 | | 1.00 - 1.00 | | 0.09 | | 1.00 | | 1.00 - 1.00 | | 0.44 | | 1.00 | | 0.99 - 1.00 | | 0.52 | | 1.00 | | 0.99 - 1.00 | | 0.64 | | |
| **Public Transit** | 1.00 | 1.00 - 1.00 | 0.17 | | 1.00 | | 1.00 - 1.00 | | 0.37 | | 1.00 | | 0.99 - 1.00 | | <0.01 | | 1.00 | | 0.99 - 1.00 | | <0.01 | | 1.00 | | 1.00 - 1.00 | | 0.05 | | |
| **Housing / Community Development** | 1.01 | 1.01 - 1.01 | <0.01 | | 1.00 | | 1.00 - 1.00 | | 0.45 | | 1.00 | | 0.99 - 1.00 | | 0.02 | | 0.99 | | 0.99 - 1.00 | | 0.02 | | 1.00 | | 0.99 - 1.00 | | 0.21 | | |
| **Other social services** | 1.01 | 0.98 - 1.04 | 0.54 | | 1.00 | | 0.98 - 1.01 | | 0.71 | | 0.99 | | 0.97 - 1.01 | | 0.17 | | 0.97 | | 0.95 - 1.00 | | 0.04 | | 0.99 | | 0.97 - 1.02 | | 0.57 | | |
| Observations | 605,732 | |  | | 605,732 | | | |  | | 605,732 | | | |  | | 605,732 | | | |  | | 605,732 | | | |  | | |
|  |  |  | |  | |  | |  | |  | |  | |  | |  | |  | |  | |  | |  | |  | |  | |
| **Abbrev**: IRR, incidence rate ratio; CI, confidence interval.  **Note:** Models adjust for beneficiary-level characteristics (age, sex, race, zip-code SDI, comorbidities), county-level characteristics (median household income, unemployment rate, PCP density, hospital beds, public health spending, market concentration, purchasing power, party vote share), and include state fixed effects. | | | | | | | | | | | | | | | | | | | | | | | | | | | | |  |

**eTable 5.** Associations Between Social Spending Quintile and Primary Care Visits, ED Visits, and ACSC Hospitalizations (County Poverty Rate Substituted for Median Household Income).

|  | **Primary Care Visits** | | | **ED Visits** | | | **PQI90** | | | **PQI91** | | | **PQI92** | | | |
| --- | --- | --- | --- | --- | --- | --- | --- | --- | --- | --- | --- | --- | --- | --- | --- | --- |
| Spending | IRR | 95% CI | p | IRR | 95% CI | p | IRR | 95% CI | p | IRR | 95% CI | p | IRR | 95% CI | p | |
| **Welfare (ref = Quintile 1)** |  |  |  |  |  |  |  |  |  |  |  |  |  |  |  | |
| Quintile 2 | 1.01 | 0.95 - 1.07 | 0.72 | 1.00 | 0.98 - 1.03 | 0.81 | 1.00 | 0.95 - 1.05 | 0.90 | 0.99 | 0.93 - 1.06 | 0.84 | 1.00 | 0.95 - 1.06 | 0.93 | |
| Quintile 3 | 1.08 | 1.01 - 1.14 | 0.02 | 0.97 | 0.95 - 1.00 | 0.08 | 0.99 | 0.95 - 1.04 | 0.78 | 0.96 | 0.90 - 1.02 | 0.22 | 1.02 | 0.96 - 1.07 | 0.58 | |
| Quintile 4 | 1.07 | 0.98 - 1.16 | 0.12 | 0.99 | 0.96 - 1.02 | 0.50 | 0.99 | 0.94 - 1.04 | 0.77 | 0.98 | 0.91 - 1.05 | 0.52 | 1.00 | 0.95 - 1.06 | 0.88 | |
| Quintile 5 | 1.06 | 0.97 - 1.16 | 0.18 | 0.99 | 0.94 - 1.04 | 0.67 | 1.03 | 0.97 - 1.09 | 0.38 | 1.04 | 0.95 - 1.14 | 0.35 | 1.02 | 0.96 - 1.09 | 0.54 | |
|  |  |  |  |  |  |  |  |  |  |  |  |  |  |  |  | |
| **Public Transit (ref = zero spending)** |  |  |  |  |  |  |  |  |  |  |  |  |  |  |  | |
| Quartile 1 | 1.08 | 1.00 - 1.17 | 0.04 | 1.03* | 1.00 - 1.06 | 0.03 | 0.99 | 0.95 - 1.03 | 0.69 | 0.98 | 0.92 - 1.04 | 0.45 | 1.00 | 0.96 - 1.05 | 0.95 | |
| Quartile 2 | 1.06 | 1.02 - 1.11 | <0.01 | 1.02 | 1.00 - 1.05 | 0.09 | 0.97 | 0.93 - 1.01 | 0.12 | 0.97 | 0.92 - 1.03 | 0.28 | 0.97 | 0.93 - 1.02 | 0.20 | |
| Quartile 3 | 0.98 | 0.93 - 1.04 | 0.54 | 1.04 | 1.01 - 1.07 | <0.01 | 0.95 | 0.92 - 0.99 | 0.01 | 0.93 | 0.87 - 0.98 | 0.01 | 0.97 | 0.93 - 1.01 | 0.13 | |
| Quartile 4 | 0.96 | 0.90 - 1.01 | 0.11 | 1.01 | 0.98 - 1.04 | 0.42 | 0.94 | 0.90 - 0.98 | <0.01 | 0.92 | 0.87 - 0.97 | <0.01 | 0.95 | 0.91 - 1.00 | 0.04 | |
|  |  |  |  |  |  |  |  |  |  |  |  |  |  |  |  | |
| **Housing (ref = Quintile 1)** |  |  |  |  |  |  |  |  |  |  |  |  |  |  |  | |
| Quintile 2 | 1.03 | 0.97 - 1.10 | 0.34 | 1.02 | 0.99 - 1.05 | 0.19 | 0.99 | 0.95 - 1.04 | 0.77 | 0.98 | 0.92 - 1.05 | 0.54 | 1.00 | 0.95 - 1.06 | 0.87 | |
| Quintile 3 | 1.14 | 1.06 - 1.22 | <0.01 | 1.00 | 0.98 - 1.03 | 0.72 | 0.97 | 0.93 - 1.02 | 0.27 | 0.96 | 0.90 - 1.03 | 0.30 | 0.98 | 0.93 - 1.04 | 0.54 | |
| Quintile 4 | 1.09 | 1.02 - 1.17 | 0.01 | 1.01 | 0.98 - 1.04 | 0.62 | 0.97 | 0.93 - 1.01 | 0.18 | 0.96 | 0.90 - 1.03 | 0.28 | 0.98 | 0.92 - 1.03 | 0.39 | |
| Quintile 5 | 1.08 | 1.00 - 1.16 | 0.04 | 0.98 | 0.95 - 1.02 | 0.31 | 0.93 | 0.89 - 0.98 | 0.01 | 0.90 | 0.84 - 0.97 | 0.01 | 0.96 | 0.90 - 1.01 | 0.12 | |
|  |  |  |  |  |  |  |  |  |  |  |  |  |  |  |  | |
| **Other social services (ref = Quintile 1)** |  |  |  |  |  |  |  |  |  |  |  |  |  |  |  | |
| Quintile 2 | 1.07 | 0.99 - 1.15 | 0.09 | 0.99 | 0.97 - 1.02 | 0.46 | 1.01 | 0.97 - 1.06 | 0.56 | 1.01 | 0.95 - 1.07 | 0.86 | 1.02 | 0.97 - 1.07 | 0.51 | |
| Quintile 3 | 1.04 | 0.99 - 1.09 | 0.10 | 1.02 | 1.00 - 1.05 | 0.06 | 1.02 | 0.98 - 1.06 | 0.34 | 0.99 | 0.94 - 1.05 | 0.82 | 1.04 | 0.99 - 1.09 | 0.14 | |
| Quintile 4 | 1.00 | 0.94 - 1.06 | 0.88 | 1.00 | 0.97 - 1.03 | 0.82 | 0.99 | 0.95 - 1.03 | 0.58 | 0.97 | 0.92 - 1.04 | 0.39 | 0.99 | 0.95 - 1.04 | 0.82 | |
| Quintile 5 | 1.04 | 0.98 - 1.10 | 0.19 | 1.01 | 0.98 - 1.04 | 0.62 | 0.98 | 0.93 - 1.02 | 0.35 | 0.96 | 0.90 - 1.02 | 0.22 | 0.99 | 0.93 - 1.04 | 0.61 | |
|  |  |  |  |  |  |  |  |  |  |  |  |  |  |  |  | |
| Observations | 605,732 | |  | 605,732 | |  | 605,732 | |  | 605,732 | |  | 605,732 | |  | |
| **Abbrev**: IRR, incidence rate ratio; CI, confidence interval.  **Note:** Models adjust for beneficiary-level characteristics (age, sex, race, zip-code SDI, comorbidities), county-level characteristics (median household income, unemployment rate, PCP density, hospital beds, public health spending, market concentration, purchasing power, party vote share), and include state fixed effects. | | | | | | | | | | | | | | | |  |

**eTable 6.** Associations Between All Spending Types and Primary Care Visits, ED Visits, and ACSC Hospitalizations (All Categories in a Single Model).

|  | **Primary Care Visits** | | | **ED Visits** | | | **PQI90** | | | **PQI91** | | | **PQI92** | | | |
| --- | --- | --- | --- | --- | --- | --- | --- | --- | --- | --- | --- | --- | --- | --- | --- | --- |
| Spending | IRR | 95% CI | p | IRR | 95% CI | p | IRR | 95% CI | p | IRR | 95% CI | p | IRR | 95% CI | p | |
| **Welfare (ref = Quintile 1)** |  |  |  |  |  |  |  |  |  |  |  |  |  |  |  | |
| Quintile 2 | 1.01 | 0.95 - 1.06 | 0.84 | 1.00 | 0.97 - 1.02 | 0.80 | 1.00 | 0.96 - 1.05 | 0.86 | 1.01 | 0.95 - 1.07 | 0.79 | 1.01 | 0.95 - 1.06 | 0.83 | |
| Quintile 3 | 1.06 | 1.00 - 1.13 | 0.06 | 0.97 | 0.94 - 1.00 | 0.06 | 1.01 | 0.96 - 1.05 | 0.78 | 0.98 | 0.92 - 1.04 | 0.55 | 1.02 | 0.97 - 1.08 | 0.39 | |
| Quintile 4 | 1.06 | 0.98 - 1.14 | 0.13 | 0.99 | 0.96 - 1.02 | 0.55 | 1.01 | 0.96 - 1.07 | 0.59 | 1.01 | 0.94 - 1.08 | 0.80 | 1.02 | 0.96 - 1.08 | 0.49 | |
| Quintile 5 | 1.09 | 1.00 - 1.19 | 0.06 | 0.99 | 0.94 - 1.04 | 0.57 | 1.05 | 0.99 - 1.12 | 0.10 | 1.08 | 0.99 - 1.18 | 0.10 | 1.04 | 0.97 - 1.11 | 0.26 | |
|  |  |  |  |  |  |  |  |  |  |  |  |  |  |  |  | |
| **Public Transit (ref = zero-spending)** |  |  |  |  |  |  |  |  |  |  |  |  |  |  |  | |
| Quartile 1 | 1.07 | 1.00 - 1.14 | 0.04 | 1.03 | 1.00 - 1.06 | 0.02 | 0.99 | 0.95 - 1.03 | 0.54 | 0.98 | 0.92 - 1.04 | 0.43 | 1.00 | 0.95 - 1.04 | 0.84 | |
| Quartile 2 | 1.06 | 1.02 - 1.11 | 0.01 | 1.02 | 1.00 - 1.05 | 0.08 | 0.97 | 0.93 - 1.01 | 0.15 | 0.98 | 0.92 - 1.03 | 0.42 | 0.97 | 0.93 - 1.01 | 0.19 | |
| Quartile 3 | 0.98 | 0.93 - 1.04 | 0.56 | 1.04 | 1.01 - 1.07 | 0.01 | 0.95 | 0.92 - 0.99 | 0.02 | 0.93 | 0.87 - 0.99 | 0.02 | 0.97 | 0.93 - 1.01 | 0.16 | |
| Quartile 4 | 0.96 | 0.92 - 1.01 | 0.14 | 1.01 | 0.98 - 1.04 | 0.54 | 0.95 | 0.91 - 0.99 | 0.02 | 0.93 | 0.88 - 0.99 | 0.02 | 0.96 | 0.91 - 1.01 | 0.09 | |
|  |  |  |  |  |  |  |  |  |  |  |  |  |  |  |  | |
| **Housing (ref = Quintile 1)** |  |  |  |  |  |  |  |  |  |  |  |  |  |  |  | |
| Quintile 2 | 1.01 | 0.95 - 1.08 | 0.74 | 1.02 | 0.99 - 1.05 | 0.29 | 0.99 | 0.95 - 1.04 | 0.70 | 0.98 | 0.92 - 1.05 | 0.58 | 1.00 | 0.95 - 1.06 | 0.98 | |
| Quintile 3 | 1.12 | 1.05 - 1.20 | <0.01 | 1.00 | 0.97 - 1.02 | 0.87 | 0.97 | 0.92 - 1.02 | 0.19 | 0.96 | 0.90 - 1.03 | 0.27 | 0.98 | 0.92 - 1.03 | 0.39 | |
| Quintile 4 | 1.11 | 1.04 - 1.19 | <0.01 | 1.00 | 0.97 - 1.03 | 0.83 | 0.97 | 0.93 - 1.02 | 0.21 | 0.97 | 0.91 - 1.04 | 0.44 | 0.97 | 0.92 - 1.03 | 0.36 | |
| Quintile 5 | 1.11 | 1.04 - 1.19 | <0.01 | 0.97 | 0.94 - 1.01 | 0.10 | 0.94 | 0.89 - 0.98 | 0.01 | 0.91 | 0.85 - 0.98 | 0.01 | 0.96 | 0.90 - 1.01 | 0.13 | |
|  |  |  |  |  |  |  |  |  |  |  |  |  |  |  |  | |
| **Other social services (ref = Quintile 1)** |  |  |  |  |  |  |  |  |  |  |  |  |  |  |  | |
| Quintile 2 | 1.04 | 0.98 - 1.10 | 0.24 | 0.99 | 0.96 - 1.02 | 0.41 | 1.01 | 0.97 - 1.06 | 0.49 | 1.01 | 0.95 - 1.07 | 0.82 | 1.02 | 0.97 - 1.07 | 0.47 | |
| Quintile 3 | 1.03 | 0.98 - 1.08 | 0.29 | 1.02 | 1.00 - 1.05 | 0.11 | 1.03 | 0.99 - 1.08 | 0.13 | 1.01 | 0.95 - 1.07 | 0.77 | 1.04 | 1.00 - 1.10 | 0.07 | |
| Quintile 4 | 0.99 | 0.94 - 1.05 | 0.79 | 1.00 | 0.97 - 1.03 | 0.84 | 1.01 | 0.97 - 1.05 | 0.77 | 1.00 | 0.94 - 1.06 | 0.91 | 1.01 | 0.96 - 1.06 | 0.73 | |
| Quintile 5 | 1.04 | 0.98 - 1.10 | 0.16 | 1.01 | 0.98 - 1.04 | 0.74 | 1.00 | 0.95 - 1.04 | 0.84 | 0.99 | 0.92 - 1.05 | 0.64 | 1.00 | 0.95 - 1.06 | 0.96 | |
|  |  |  |  |  |  |  |  |  |  |  |  |  |  |  |  | |
| Observations | 605,732 | |  | 605,732 | |  | 605,732 | |  | 605,732 | |  | 605,732 | |  | |
| **Abbrev**: IRR, incidence rate ratio; CI, confidence interval.  **Note:** Models adjust for beneficiary-level characteristics (age, sex, race, zip-code SDI, comorbidities), county-level characteristics (median household income, unemployment rate, PCP density, hospital beds, public health spending, market concentration, purchasing power, party vote share), and include state fixed effects. | | | | | | | | | | | | | | | |  |
